# Letter from H. O. Hitchcock, M. D., Kalamazoo, Mich.

**Published:** 1867-12

**Authors:** H. O. Hitchcock

**Affiliations:** Kalamazoo, Mich.


KALAMAZOO, MICH., *November 14, 1867.*

DR. J. ADAMS ALLEN, *Editor Chicago Medical Journal:*

*Dear Sir:*—So many persons have written me enquiring about the source of Propolis, and whether it is identified with bee-bread, I would thank you to state in the next issue of the Journal that Propolis is found in old bee-hives. It is not the same as the bee-bread but is very different. It is used by the bees as a sort of glue or cement with which they smear over any rough places in the hive; fill up any holes, and cement the comb to the sides of the hive. It can be readily distinguished, and easily collected from any old bee-hive. Its vegetable origin and source is not very definitely known, but is probably from the buds of certain trees, such as the birches, the hickory, and especially the tree known as the balm of Gilead.

Yours, Truly, H. O. HITCHCOCK.

